# Supplementary material for: The impact of diabetes mellitus medication on the incidence of endogenous endophthalmitis
Source: PLoS One. 2020 Jan 10;15(1):e0227442. doi: 10.1371/journal.pone.0227442 (PMC6953870; doi:10.1371/journal.pone.0227442)
Supplement: S1 Table — (DOC) [file pone.0227442.s001.doc]

**S1 Table.** Characteristics of the study sample at baseline.

| **Medication** | **Total** | | **With** | | **Without** | | **P** |
| --- | --- | --- | --- | --- | --- | --- | --- |
| **Variables** | **n** | **%** | **n** | **%** | **n** | **%** |
| **Total** | 121,800 |  | 24,360 | 20.00 | 97,440 | 80.00 |  |
| **Gender** |  |  |  |  |  |  | 0.999^†^ |
| Male | 64,572 | 53.01 | 12,914 | 53.01 | 51,658 | 53.02 |  |
| Female | 57,228 | 46.99 | 11,446 | 46.99 | 45,782 | 46.98 |  |
| **Age (years)** | 62.36±15.12 | | 62.24±13.26 | | 62.39±15.55 | | 0.167^§^ |
| **Age group (years)** |  |  |  |  |  |  | 0.999^†^ |
| 18-44 | 12,820 | 10.53 | 2,564 | 10.53 | 10,256 | 10.53 |  |
| 45-64 | 53,145 | 43.63 | 10,629 | 43.63 | 42,516 | 43.63 |  |
| ≧65 | 55,835 | 45.84 | 11,167 | 45.84 | 44,668 | 45.84 |  |
| **Liver abscess** |  |  |  |  |  |  | 0.12^‡^ |
| With | 1,366 | 1.12 | 291 | 1.19 | 1,075 | 1.10 |  |
| **HT** |  |  |  |  |  |  | 0.004^†^ |
| With | 49,538 | 40.67 | 9,727 | 39.93 | 39,811 | 40.86 |  |
| **Depression** |  |  |  |  |  |  | 0.011^‡^ |
| With | 921 | 0.76 | 156 | 0.64 | 765 | 0.79 |  |
| **Anxiety** |  |  |  |  |  |  | 0.23^‡^ |
| With | 485 | 0.40 | 90 | 0.37 | 395 | 0.41 |  |
| **Renal disease** |  |  |  |  |  |  | <0.001^†^ |
| With | 7,132 | 5.86 | 1,315 | 5.40 | 5,817 | 5.97 |  |
| **Hyperlipidemia** |  |  |  |  |  |  | <0.001^†^ |
| With | 11,317 | 9.29 | 2,471 | 10.14 | 8,846 | 9.08 |  |
| **Thyrotoxicosis** |  |  |  |  |  |  | 0.021^†^ |
| With | 2,809 | 2.31 | 605 | 2.48 | 2,204 | 2.26 |  |
| **Septicemia** |  |  |  |  |  |  | 0.091^†^ |
| With | 4,875 | 4.00 | 938 | 3.85 | 3,937 | 4.04 |  |
| **Pneumonia** |  |  |  |  |  |  | 0.057^†^ |
| With | 8,447 | 6.94 | 1,633 | 6.70 | 6,814 | 6.99 |  |
| **Liver disease** |  |  |  |  |  |  | 0.455^†^ |
| With | 7,878 | 6.47 | 1,580 | 6.49 | 6,298 | 6.46 |  |
| **Tumor** |  |  |  |  |  |  | <0.001^†^ |
| With | 9,308 | 7.64 | 1,630 | 6.69 | 7,678 | 7.88 |  |
| **CCI_R** | 0.22±0.57 | | 0.22±0.58 | | 0.21±0.57 | | 0.079^§^ |
| P-values were determined using chi-square tests (indicated by ^†^) or the Fisher’s exact test (indicated by ^‡^) for categorical variables and t-tests (indicated by ^§^) for continuous variables | | | | | | | |
