# Supplementary material for: The impact of diabetes mellitus medication on the incidence of endogenous endophthalmitis
Source: PLoS One. 2020 Jan 10;15(1):e0227442. doi: 10.1371/journal.pone.0227442 (PMC6953870; doi:10.1371/journal.pone.0227442)
Supplement: S2 Table — (DOC) [file pone.0227442.s002.doc]

**S2 Table.** Characteristics of the study sample at the end of the follow-up.

| **Medication** | **Total** | | **With** | | **Without** | | **P** |
| --- | --- | --- | --- | --- | --- | --- | --- |
| **Variables** | **n** | **%** | **n** | **%** | **n** | **%** |
| **Total** | 121,800 |  | 24,360 | 20.00 | 97,440 | 80.00 |  |
| **Endogenous endophthalmitis** |  |  |  |  |  |  | 0.043^‡^ |
| Without | 121,674 | 99.90 | 24,343 | 99.93 | 97,331 | 99.89 |  |
| With | 126 | 0.10 | 17 | 0.07 | 109 | 0.11 |  |
| **Gender** |  |  |  |  |  |  | 0.999^†^ |
| Male | 64,572 | 53.01 | 12,914 | 53.01 | 51,658 | 53.02 |  |
| Female | 57,228 | 46.99 | 11,446 | 46.99 | 45,782 | 46.98 |  |
| **Age (years)** | 67.50±13.72 | | 67.89±13.66 | | 67.40±13.73 | | <0.001^§^ |
| **Age group (years)** |  |  |  |  |  |  | <0.001^†^ |
| 18-44 | 10,649 | 8.74 | 2,000 | 8.21 | 8,649 | 8.88 |  |
| 45-64 | 48,976 | 40.21 | 9,486 | 38.94 | 39,490 | 40.53 |  |
| ≧65 | 62,175 | 51.05 | 12,874 | 52.85 | 49,301 | 50.60 |  |
| **Liver abscess** |  |  |  |  |  |  | 0.193^‡^ |
| With | 2,246 | 1.84 | 466 | 1.91 | 1,780 | 1.83 |  |
| **HT** |  |  |  |  |  |  | 0.295^†^ |
| With | 46,230 | 37.96 | 9,283 | 38.11 | 36,947 | 37.92 |  |
| **Depression** |  |  |  |  |  |  | 0.036^‡^ |
| With | 1,027 | 0.84 | 182 | 0.75 | 845 | 0.87 |  |
| **Anxiety** |  |  |  |  |  |  | 0.012^‡^ |
| With | 409 | 0.34 | 63 | 0.26 | 346 | 0.36 |  |
| **Renal disease** |  |  |  |  |  |  | 0.253^†^ |
| With | 9,688 | 7.95 | 1,912 | 7.85 | 7,776 | 7.98 |  |
| **Hyperlipidemia** |  |  |  |  |  |  | <0.001^†^ |
| With | 9,253 | 7.60 | 2,044 | 8.39 | 7,209 | 7.40 |  |
| **Thyrotoxicosis** |  |  |  |  |  |  | 0.211^‡^ |
| With | 2,316 | 1.90 | 479 | 1.97 | 1,837 | 1.89 |  |
| **Septicemia** |  |  |  |  |  |  | 0.018^†^ |
| With | 11,012 | 9.04 | 2,287 | 9.39 | 8,725 | 8.95 |  |
| **Pneumonia** |  |  |  |  |  |  | 0.028^†^ |
| With | 13,189 | 10.83 | 2,721 | 11.17 | 10,468 | 10.74 |  |
| **Liver disease** |  |  |  |  |  |  | 0.275^†^ |
| With | 7,553 | 6.20 | 1,490 | 6.12 | 6,063 | 6.22 |  |
| **Tumor** |  |  |  |  |  |  | <0.001^†^ |
| With | 13,349 | 10.96 | 2,520 | 10.34 | 10,829 | 11.11 |  |
| **CCI_R** | 0.26±0.67 | | 0.26±0.66 | | 0.26±0.68 | | 0.528^§^ |
| P-values were determined using chi-square tests (indicated by ^†^) or the Fisher’s exact test (indicated by ^‡^) for categorical variables and t-tests (indicated by ^§^) for continuous variables. | | | | | | | |

Aged 18 and stratified by medication use with the log-rank test.
