# Supplementary material for: The impact of diabetes mellitus medication on the incidence of endogenous endophthalmitis
Source: PLoS One. 2020 Jan 10;15(1):e0227442. doi: 10.1371/journal.pone.0227442 (PMC6953870; doi:10.1371/journal.pone.0227442)
Supplement: Attachment — Submitted filename: Response to Reviewers-PONE 20191118.docx [file pone.0227442.s003.docx]

PLOS ONE

**Editor-in-Chief,**

November 11, 2019

Dear Editor-in-Chief,

We very much appreciate the comments and critiques from the reviewers regarding Manuscript ID PONE-D-19-23998, entitled "The impact of diabetes mellitus medication on the incidence of endogenous endophthalmitis". We have performed the additional work requested by the reviewers to improve our manuscript. We have also written point-by-point responses to the reviewers’ comments. Overall, the manuscript has been substantially revised to reflect each of the reviewers’ suggestions. All coauthors have read and approved this manuscript and agreed to fully transfer its copyright to ***PLOS ONE.*** I sign for and accept responsibility for this material on behalf of all coauthors. We hope that these changes will make the paper more impactful and that it can be published in your journal in the near future.

Thank you for your time and consideration.

Sincerely,

Ke-Hung Chien, MD

Tzu-Heng Weng, MD

Department of Ophthalmology, Tri-Service General Hospital, National Defense Medical Center, Taipei, Taiwan; No. 325, Section 2, Cheng-Kung Road, Neihu District, Taipei City, 11490, Taiwan, Republic of China.

**Answers to the comments from the Reviewer(s) and Editor(s)**

**Reviewer #1**

1. **The first is that no information was provided on patient diagnosis such as A1C levels or whether the patients were type 1 or 2.**

Reply: Thank you for your comments. NHIRD does not offer detailed clinical information regarding clinical manifestations or disease severity, and laboratory findings are lacking, especially HbA1c data, which are particularly essential in diagnosing DM and monitoring disease progression. This is indeed a limitation, which we have stated at the end of the Discussion section.

Please refer to the **Discussion** section (lines 302-306) in the revised manuscript. The changes are highlighted in red.

We also wish to conduct another retrospective study in the future to investigate the relationship between HbA1c and EE incidence.

The DM patients in this study were identified according to the ICD-9 code 250, including type 1 and 2 DM patients. This is stated in the Methods section.

Please refer to the **Methods** section (lines 87-91) in the revised manuscript.

2. **It also is not clear why so many patients with diabetes were not being treated. Were they all under dietary control for diabetes, or were they without medication due to lack of compliance or lack of medical care?**

Reply: Thank you for your comments. In the present study, we identified 26,360 patients who were newly diagnosed with and treated for DM in 2000 to 2010. The comparison group included individuals who were newly diagnosed with DM and were not treated with medication during the same time period. The comparison group was presumed to have less severe DM initially and was therefore treated with lifestyle modification and dietary control. If the patients had poor medication compliance, which would mean that medication had been prescribed but the patients did not take it, then these cases were not classified into the comparison group. This statement has also been added to the Methods and Results sections according to your suggestion.

Please refer to the **Methods** (lines 88) **and Results** (lines 128) sections in the revised manuscript. The changes are highlighted in red.

3. **The authors were not clear on their rationale for performing this study. Indeed, diabetes mellitus is an underlying risk factor for developing endogenous endophthalmitis, however they were vague in describing the mechanism for this. Another group has demonstrated that this correlation is likely due to the break down of the blood retinal barrier during diabetes development and progression to diabetic rertinopathy, and likely has little to do with keratitis.**

Reply: Thank you for your comments and suggestions. According to your suggestion, we have briefly discussed the mechanisms of endogenous endophthalmitis developed in DM in the Discussion section.

Please refer to the **Discussion** section (lines 203-210) in the revised manuscript. The changes are highlighted in red.
